# Transcriptional profiling of the pea shoot apical meristem reveals processes underlying its function and maintenance

**DOI:** 10.1186/1471-2229-8-73

**Published:** 2008-06-30

**Authors:** Chui E Wong, Prem L Bhalla, Harald Ottenhof, Mohan B Singh

**Affiliations:** 1 Plant Molecular Biology and Biotechnology laboratory, Australian Research Centre of Excellence for Integrative Legume Research, Faculty of Land and Food Resources, The University of Melbourne, Parkville, Victoria 3010, Australia

## Abstract

**Background:**

Despite the importance of the shoot apical meristem (SAM) in plant development and organ formation, our understanding of the molecular mechanisms controlling its function is limited. Genomic tools have the potential to unravel the molecular mysteries of the SAM, and legume systems are increasingly being used in plant-development studies owing to their unique characteristics such as nitrogen fixation, secondary metabolism, and pod development. Garden pea (*Pisum sativum*) is a well-established classic model species for genetics studies that has been used since the Mendel era. In addition, the availability of a plethora of developmental mutants makes pea an ideal crop legume for genomics studies. This study aims to utilise genomics tools in isolating genes that play potential roles in the regulation of SAM activity.

**Results:**

In order to identify genes that are differentially expressed in the SAM, we generated 2735 ESTs from three cDNA libraries derived from freshly micro-dissected SAMs from 10-day-old garden peas (*Pisum sativum *cv Torsdag). Custom-designed oligonucleotide arrays were used to compare the transcriptional profiles of pea SAMs and non-meristematic tissues. A total of 184 and 175 transcripts were significantly up- or down-regulated in the pea SAM, respectively. As expected, close to 61% of the transcripts down-regulated in the SAM were found in the public database, whereas sequences from the same source only comprised 12% of the genes that were expressed at higher levels in the SAM. This highlights the under-representation of transcripts from the meristematic tissues in the current public pea protein database, and demonstrates the utility of our SAM EST collection as an essential genetic resource for revealing further information on the regulation of this developmental process. In addition to unknowns, many of the up-regulated transcripts are known to encode products associated with cell division and proliferation, epigenetic regulation, auxin-mediated responses and microRNA regulation.

**Conclusion:**

The presented data provide a picture of the transcriptional profile of the pea SAM, and reveal possible roles of differentially expressed transcripts in meristem function and maintenance.

## Background

Organ formation is not limited to embryonic development, but can occur throughout the lifetime of a plant. The potential to develop new organs post-embryonically is attributed to meristems located at the growing tips of the plants, with the root apical meristem generating the underground part of the plant and the shoot apical meristem (SAM) giving rise to the entire shoot system after seed germination.

Like its root counterpart, the SAM contains a pool of pluripotent stem cells that can self-maintain as well as produce the cells that can differentiate into multiple cell and tissue types [reviewed in [1]]. While lateral organs such as the leaves are initiated from the peripheral regions of the SAM, the basal regions of the SAM contribute to the formation of the stem. The stem cells of the SAM must thus replenish areas where cells have been recruited and at the same time maintain the population of stem cells. This is generally attributed to an active process of communication among neighbouring SAM cells in the microenvironment of the stem cells [2-4].

Elegant genetic work carried out in the model plant, *Arabidopsis thaliana*, has enhanced our understanding of this vital developmental process [reviewed by [5]]. This is exemplified by the identification in *Arabidopsis *of WUSCHEL (WUS), a homeodomain transcription factor essential for maintaining the pools of stem cells in an undifferentiated state [6], and the *CLAVATA *group of genes that act together to restrict the proliferation of stem cells [7]. While the *Arabidopsis *genus provides invaluable model plants for enhancing our understanding of plant biology, it does not represent all the diverse developmental, environmental and physiological processes operating in the plant kingdom. There thus remains a need to extend the knowledge gained to other plant species especially crop plants.

Applying modern genomics research techniques to improving crops requires new knowledge and the development of new genomics resources. Legume species belonging to the family Fabaceae are cultivated for seeds rich in proteins, and represent important components of the diet in many parts of the world, especially pea, lentil and soybean. Legumes have attracted the attention of biologists because of their unique characteristics such as nitrogen fixation, secondary metabolism and pod development, and these are among the various processes that cannot be studied in *Arabidopsis *species. On the other hand, garden pea (*Pisum sativum*) has been a classic model species used in genetics [8,9] and plant-development studies. Its extensive use in studies of flowering initiation and development has provided important insights into this transition process [10-13]. In addition, the availability of various developmental and flowering pea mutants [11,14-18] makes this tractable for genomics studies.

In this study, we applied a transcriptomics approach to investigate the gene expression profiles associated with the SAM of the garden pea, an agriculturally and commercially important model legume. We also investigated the use of micro-dissected SAMs in unravelling the transcriptome profile of the SAM. By identifying genes that exhibit differential expression between SAMs and non-meristematic tissues (NM), we aimed to elucidate the transcriptional signature of the SAM and thereby identify genes that might play important roles in regulating SAM activity.

To this end, three directional cDNA libraries were constructed using SAMs that were carefully micro-dissected from garden peas. These libraries comprised the standard cDNA library plus a normalized library and a subtracted library, in order to increase the likelihood of recovering rare cDNAs, allowing the sampling of the wide diversity of genes expressed in the pea SAM. The EST sequences derived from these libraries were used in the subsequent design of a CombiMatrix CustomArray™ 4 × 2 K oligonucleotide array that was representative of the gene content of the SAM.

In this paper, we present the EST and transcriptional profiling data from this genomics project. The transcriptional profiling experiment represents the first analysis of genes that exhibit differential expression between the pea SAM and NM. The data reveal that transcripts putatively annotated as being associated with cell division and proliferation, epigenetic regulation, auxin-mediated responses and microRNA (miRNA) regulation are more abundant in the SAM than in the NM. In contrast, sequences related to photosynthesis, abiotic or biotic stress responses, reactive oxygen species (ROS) homeostasis and general cell-wall maintenance are down-regulated in the SAM.

## Results and Discussion

### Features of generated ESTs

A total of 3000 clones from three cDNA libraries were single-pass sequenced from their 5' ends. Sequence cleaning processes as outlined in the Materials and Methods yielded 2735 ESTs. These sequences had an average trimmed length of 519 base pairs and were assembled into 348 clusters and 1332 singlets, resulting in the final annotation of 1686 unigenes. Clusters ranged in membership from 67 ESTs (one) to two ESTs (253). The redundancy levels were 15.0%, 20.3% and 62.8% within the normalized, standard and subtracted libraries, respectively. A high redundancy level of cDNA libraries constructed using a similar subtraction method from *Thellungiella *plants under abiotic stress conditions has also been observed previously [19].

The translated products of the 1686 unigenes were searched against the non-redundant protein database provided by GenBank [20] to putatively assign their functions. At the time of writing, 918 (54.4%) unigenes showed significant similarity (*E *value cut-off of 10^-5^) to genes of known or putative function, whereas 549 (32.6%) ESTs were assigned to transcripts with unknown function, which includes hypothetical genes predicted in genomes of model organisms (Table 1). The remaining 219 ESTs (13.0%) had no homologues in the public protein sequence database. A further BLASTN search against GenBank EST collections revealed that 62 of the 219 ESTs were likely to be novel sequences.

**Table 1:** A summary of the results obtained from BLASTX search against the NCBI (nr) protein database with expect value cut-off at 1e-5.

**Category**	**Number of unique ESTs**
Match to genes with known or putative function	918 (54.4%)
Match to unknown or hypothetical genes	549 (32.6%)
No hits found	219 (13.0%)

Total	1686

### Overview of putative genes represented in SAM-derived cDNA libraries

**Figure 1:**
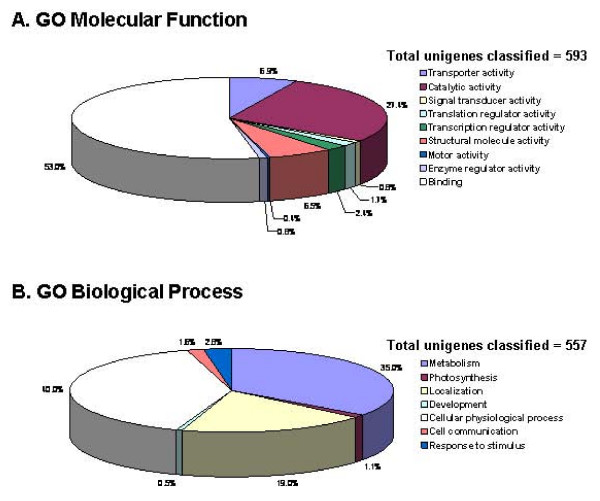
**Categorization of SAM unigenes according to the Gene Ontology (GO).** Unigenes with a BLASTX score of < 10^-5 ^were classified using the BLAST2GO automated system [21]. Note that a single gene can be assigned to more than one category in the GO classification system.

More than 50% of the SAM unigenes could be assigned as genes with a known or putative function based on sequence similarity. However, the lack of information on the encoded products meant that many of these transcripts could not be functionally categorized according to the Gene Ontology Consortium (GO). Using BLAST2GO [21], we successfully classified 593 and 557 unigenes in terms of GO molecular functions (Figure 1A) and biological processes (Figure 1B), respectively. A single gene product might be assigned to more than one GO term, and hence the total number of GO mappings in each of the ontologies exceeded the number of ESTs.

The successfully classified unigenes cover a broad range of GO functional categories (Figure 1). Under a molecular-function classification, most of the genes (53%) were assigned to the "binding" class (Figure 1A). This class includes sequences with putative involvement in mainly nucleic acid binding, a substantial number of which are predicted to encode transcription factors that are known to be essential to the regulation of plant development. Unigenes predicted to encode histone subunits and histone-modification proteins, chromatin remodelling factors and DNA methyltransferases represent another group of sequences linked to nucleic acid binding.

To investigate the different types of transcription-factor families represented by our EST libraries, a search using the best-matching *Arabidopsis *locus for SAM unigenes (based on a BLASTX search against the TAIR *Arabidopsis *protein database) was performed at the *Arabidopsis *Gene Regulatory Information Server [22]. There are 50 families of transcription factors currently listed in the database, 19 of these are represented by the SAM unigenes (Table 2). Six members of the family of homeobox transcription factors that play key roles in the regulation of development may represent interesting candidate genes for further studies.

**Table 2:** ESTs related to the ontology of nucleic acid binding.

**SAM Clone**	**Family**	**Matching Arabidopsis Locus^a^**
EX568781, EX569978	CCAAT-HAP5	At1g07980, At1g08970
EX569532	MYB-Related	At1g09770
EX569985, EX569919	TUB	At1g16070, At2g18280
EX569797, EX569977	GRAS	At1g21450, At5g66770
EX569951	AP2-EREBP	At1g28360
EX571097	NAC	At1g28470
EX570087, EX570238, EX569491	ARF	At1g30330, At4g23980, At5g20730
EX571252, EX569634, EX570163, EX569945	WRKY	At1g30650, At2g03340, At2g47260, At2g24570
EX570518, EX569818	bZIP	At1g42990, At4g34590
EX570905, EX569205, EX570291, EX568744	C2H2	At1g55110, At1g75710, At2g23740, At2g27100
EX568999, EX571110, EX570832, EX571147, EX569211, EX570070	Homeobox	At1g62360, At2g27990, At4g36870, At4g40060, At4g32980, At5g03790
EX570236	G2-like	At1g69580
EX569752, EX569559	ARID	At1g76110, At1g76510
EX571125	BHLH	At2g27230
EX568890	CCAAT-HAP3	At2g37060
EX569425	MADS	At2g45660
EX569354	C2C2-Gata	At3g06740
EX569807	SBP	At3g60030

Arabidopsis transcription factor family represented by the shoot apical meristem (SAM) unigenes. The matching Arabidopsis locus for each clone is indicated.

a. Annotation is based on the best BLASTX match against TAIR Arabidopsis protein database (Evalue < 1e-5)

In the biological-processes category, 40% and 35% of the unigenes were involved with cellular physiological processes and metabolism, respectively (Figure 1B). The former contains gene products that play an important role in cell organization and biogenesis, while the latter has sequences related mainly to protein metabolism. The sequences relevant to protein metabolism ranged from those associated with protein biosynthesis, such as different ribosomal subunits, to those that modify and degrade proteins, including various sequences involved in the ubiquitin-proteasome pathway.

### Detection of differentially expressed transcripts in the SAM using the pea 2 K array

**Figure 2:**
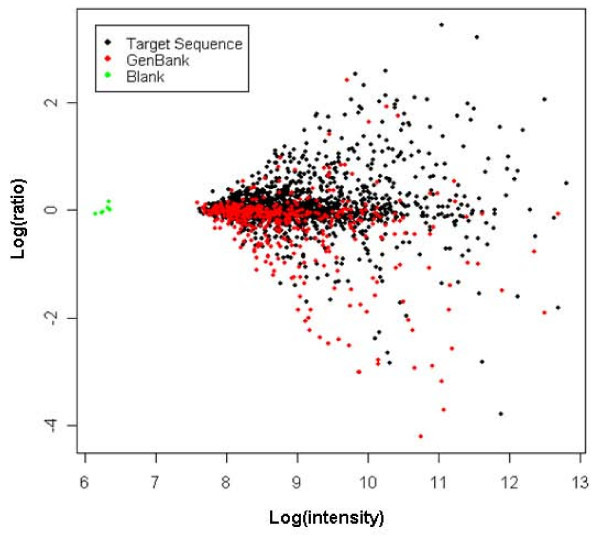
**Results of transcript profiling experiments using the custom designed pea SAM Combimatrix 2 K chip.** Expression profiles of SAM were compared with those of NM tissues. Average plot were generated from the experimental data of four independent biological replicates after normalization (see Materials and Methods for details). Black dotsrepresents data generated from the sequences derived from this study while red applies to those from the GenBank. Green corresponds to empty spot on the array. log(ratio) = log_10_(I_SAM_/I_NM_); log(intensity) = 0.5 log_10_(I_SAM_.I_NM_) where I_SAM _and I_NM _are signal intensities for a transcript in the SAM or the NM tissues, respectively.

An oligonucleotide microarray has been developed using our EST resource and about 500 pea sequences randomly retrieved from the GenBank pea protein database, which contains approximately 2000 entries (see Materials and Methods). We utilized this array to compare the transcriptional profiles of the SAM and NM. Four independent replications of balanced-block-design dual-label experiments were performed [Materials and methods, [23]] and the resulting data were depicted in Figure 2.

**Figure 3:**
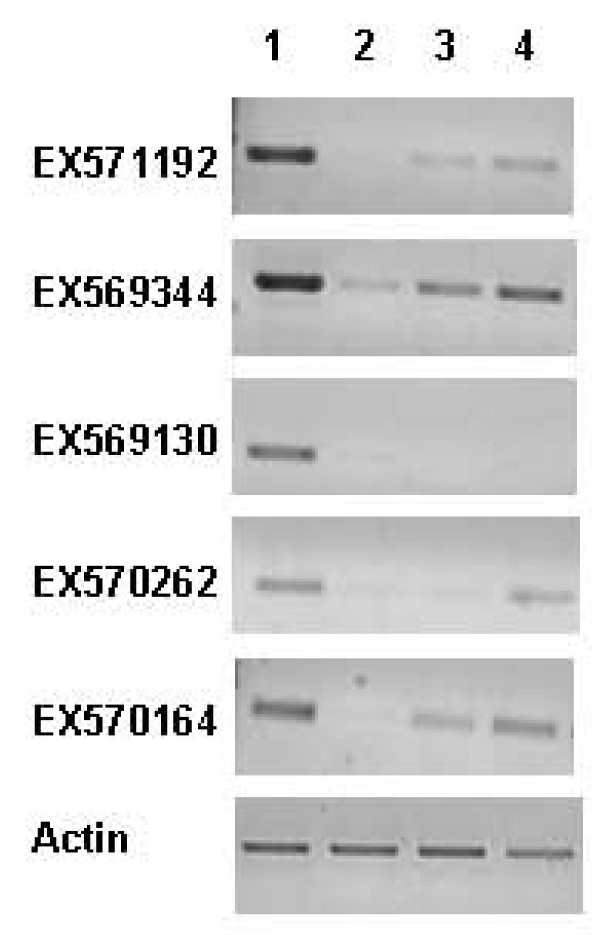
**Verification of microarray data using RT-PCR analysis.** RT-PCR analysis was carried out under linear amplification conditions for five randomly selected transcripts as indicated. The *actin *gene was used as an internal control. 1, SAM; 2, Leaf; 3, Stem; 4, Root.

Differentially expressed genes that were detected using LimmaGUI [24] (at an adjusted probability value of < 0.05) were subjected to further selection based on the relative change, with up- and down-regulated transcripts defined using cut-offs of greater than 1.3-fold or less than 0.7-fold, respectively. Based on these criteria, we identified 184 and 175 transcripts that were significantly up- or down-regulated in the pea SAM relative to the NM. These transcripts were annotated based on the best BLASTX match, and exhibited changes relative to the NM of 0.1- to 10.7-fold (see Additional File 1 &2). The change of 0.1-fold was for a gene encoding a type 1 metallothionein (AB176564), while that of 10.7-fold was for a sequence annotated as *vegetative lectin *(AAA33691). A study of similar genes encoding type 1 metallothionein in cotton revealed their abundant (although not exclusive) expression in the root [25]. Meanwhile, the high expression of a sequence encoding vegetative lectin in the pea apex has been observed [26]. These and other studies listed in Table 3 provide independent verification of our microarray data. We also performed RT-PCR analysis on five selected transcripts and as shown in Figure 3, the outcome is generally in good agreement with the microarray data.

**Table 3:** Differentially-regulated transcripts with corresponding orthologues known to be highly expressed in shoot apical meristems (SAMs) or non-meristem (NM) tissues.

**Probe ID^a^**	**Annotation**	**Fold change**	**References**
AB176564	Metallothionein	0.1	Hudspeth et al., 1996
AF029242	Dormancy associated gene 1 (DRM1)	0.4	Stafstrom et al., 1998
EX570325	MERISTEMATIC RECEPTOR-LIKE KINASE	1.4	Fujita et al., 2003
EX568912	F-box protein (STAMINA PISTILLOIDA)	1.8	Taylor et al., 2001
EX570203	Protodermal factor 1	1.8	Abe et al., 2001
EX568701, EX571325, EX569084, EX570634	Histone subunits	2.0	References in Meshi et al., 2000
EX570428	Mini-chromosome maintenance proteins	2.2	Stevens et al., 2002
EX570270, AB008186	Proliferating cell nuclear antigen	4.8, 3.8	Kosugi et al., 1991
AB031227	PsAD1	5.3	Madoka &Mori, 2000
EX570531	Vegetative lectin	10.7	Dobres & Thompson, 1988

The data reported is in good agreement with the transcript profiling data.

a. GenBank accession number that begins with EX is derived from this study.

**Figure 4:**
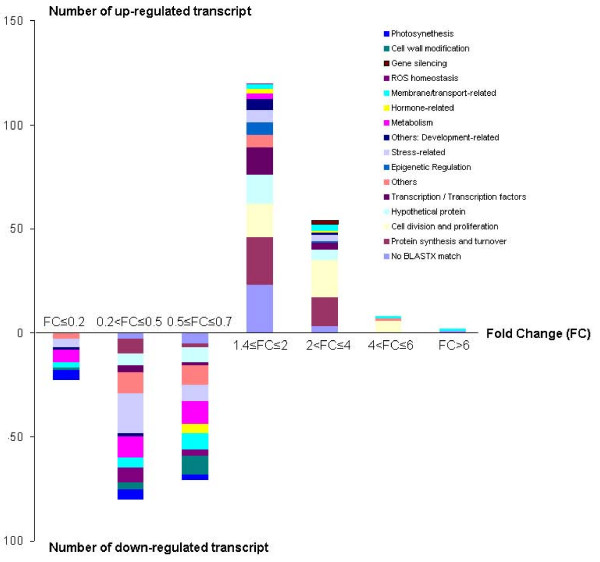
**Functional categories of transcripts differentially expressed in the pea SAM.** The number of genes differentially expressed between the pea SAM and NM (p < 0.05) with changes in expression higher than indicated cut offs of the fold change are plotted with different functional categories highlighted by colour coding. Transcripts corresponding to cell division and proliferation, epigenetic regulation, and gene silencing are among the up-regulated categories while stress response and the metabolism class of genes are in down-regulated categories.

Further comparison of the functional categories identified to be differentially regulated in maize data [25] and this study (Figure 4) revealed that transcripts associated with the categories of transcription, chromatin and cell division are highly represented in the list of up-regulated genes, while sequences categorized as being related to metabolism, stress and photosynthesis are down-regulated in the SAM. However, our data also highlight differences between the transcriptional repertoires of the monocot SAM [maize, [27]] and the dicot SAM (pea, present study). In particular, the high retrotransposon-related transcriptional activity reported in maize [27] does not appear to be a conserved feature of the SAM, since it was not present in the pea SAM ESTs.

Since the SAM we investigated consists of distinct functional zones, the averaging of signals across the whole SAM probably attenuated signals associated with any given stem-cell region. However, our experiment showed that a sequence annotated as encoding PROTODERMAL FACTOR 1 was up-regulated 1.8-fold in the SAM relative to the NM (Table 3). An *Arabidopsis *counterpart gene has been found to be exclusively expressed in the outermost (L1) layer of the SAM [28]. This indicates the ability to identify transcripts that are specifically expressed in certain domains of the SAM, though the magnitude of the relative change might have been reduced and low abundance transcript could have been missed detection.

A closer inspection of the differentially expressed transcripts revealed that about 60% of the identified down-regulated sequences were derived from GenBank (Table 4). In contrast, only 12% of the genes with higher expression in SAMs were retrieved from GenBank, while the other up-regulated ESTs were from clones derived from our library collection. This is not surprising since the sequences from GenBank were generated primarily from tissues other than the SAM, whereas our EST collection was derived from dissected SAMs. This indicates the significance of constructing a library from specialized tissues and further suggests the utility of our EST collection as a valuable resource in studying the molecular processes underlying the functions of the plant meristem. Meanwhile, 30% of the differentially expressed genes detected were not identified, consisting of genes annotated as hypothetical or expressed protein as well as sequences that have no BLASTX matches in the public database. This list of unknowns could represent intriguing candidate genes for functional analysis.

**Table 4:** Representative transcripts that are detected to be significantly up-regulated in the pea shoot apical meristem (SAM) in comparison to the non-meristem (NM) tissues.

**Probe^a^**	**Fold Change**	**Annotation^b^**
**Cell division and proliferation**		
EX570197	2.7	High mobility group protein (HMGI/Y)
EX571067	1.6	SAR-DNA binding protein 2
EX571065	1.7	SAR-DNA binding protein 1
EX570428	1.8	Mini-chromosome maintenance protein 6
EX569064	2.0	Small nuclear ribonucleoprotein associated protein B
EX568701	2.0	Histone H1
EX568991	2.1	Delta DNA polymerase
EX570486	2.1	Helicase
EX570226	2.2	Phosphoesterase
EX571345	3.3	Germinal histone H4
EX569084	3.7	Histone H2a.1
EX570634	4.2	Histone H3
EX570270	4.8	Proliferating cell nuclear antigen 2
EX568755	5.9	Histone H4
AB008188	1.9	Cyclin D3.1 protein
EX570164	1.8	Mitotic cyclin B1-1
EX570388	1.5	Cell cycle protein kinase
**Protein synthesis and turnover**		
EX569344	1.4	Transducin family of protein (SLOW WALKER 1)
EX570594	1.9	60S ribosomal protein L18a
EX569298	1.9	Ribosomal protein L30
EX569956	2.0	40S ribosomal protein S18
EX568807	2.1	Fibrillarin
EX570044	2.1	60S ribosomal protein L44
EX570565	2.1	Ribosomal protein L23
EX568742	2.2	Ribosomal protein L24
EX570468	2.2	60S ribosomal protein
EX568861	2.4	40S ribosomal protein S17
EX569048	2.6	Ribosomal protein S2
EX568908	2.9	Ribosomal protein S15-like
EX568954	3.0	Ribosomal protein S3
AB021873	3.2	Ribosome sedimenting protein
EX570400	5.0	Ribosomal protein S4
EX569708	2.3	Ubiquitin extension protein 2
EX568912	1.8	F-box protein (STAMINA PISTILLOIDA)
EX570030	1.9	Chaperonin
EX568849	1.6	Elongation factor 1-beta/EF-1-beta
EX570437	1.4	Cyclophilin
EX568710	1.4	Eukaryotic initiation factor
EX570401	1.4	Translation initiation protein
**Transcription factors or hormonal regulation**		
EX569978	1.5	Putative Hap5 transcription factor
EX570025	1.5	YABBY family transcription factor
EX570238	1.5	Auxin response factor 9
EX570380	3.4	Putative transcriptional co-activator (KIWI)
EX571172	1.6	Transcription factor
EX570262	2.1	Argonaute protein
EX570187	2.9	Indole-3-acetic acid amido synthetase (DWARF IN LIGHT 1)
AF325121	1.6	Brassinosteroid biosynthetic protein
**Epigenetic regulation-related**		
EX568764	1.4	Chromomethylase
AF034419	1.4	DNA methyltransferase
EX569897	1.6	H3 lysine-9 specific SUVH4
EX570366	1.9	SWI/SNF-like ATPase subunits, DDM1
EX570306	2.0	WD-40 repeat protein (MSI3)
DQ026703	1.5	WD-40 repeat protein (MSI1)
**Other developmental regulation-related**		
EX569130	2.0	Mandelonitrile lyase family of FAD containing oxidoreductases (HOTHEAD)
AY343326	1.5	Late-flowering gene
**Unclassified/Unknown/No BLASTX match**		
EX571192	9.0	No BLASTX match
EX569831	3.3	Proline-rich protein
EX570071	3.7	Hypothetical protein
EX571142	3.6	Pathogenesis-related group 5 protein
EX571013	2.8	No BLASTX match
EX570256	2.4	Unnamed protein product
EX569233	2.1	Serine/threonine dehydratase
EX569274	4.5	Lipid transfer protein
EX570925	3.1	Kunitz inhibitor ST1-like
AB032830	3.3	Endo-1,4-beta-glucanase
EX570531	10.7	Vegetative lectin
EX569068	2.4	Expressed protein
EX569663	2.1	Expressed protein
EX570209	2.2	Hypothetical protein
EX570712	2.0	Hypothetical protein
EX570576	1.9	Cytochrome P450 monoxygenase(CYP78A8)
EX569596	1.8	No BLASTX match
EX570325	1.5	MERISTEMATIC RECEPTOR-LIKE KINASE 2
EX570536	2.4	Unknown protein
EX570361	1.5	No BLASTX match
EX570202	1.4	No BLASTX match
EX570695	1.4	No BLASTX match
EX569023	1.6	No BLASTX match
EX570276	1.5	No BLASTX match
EX569420	1.4	No BLASTX match
EX568900	1.6	No BLASTX match

a, Probe with GenBank accession number that begins with EX is derived from this study.

b. Annotation is based on the expect value of BLASTX hit that is set at 1e-5

### Cell division and proliferation in the SAM

Putative functions could be assigned to 116 of the up-regulated transcripts based on protein sequence similarity. Manual inspection of the corresponding transcripts revealed a high representation of ESTs predicted to encode proteins associated with cell division and proliferation (Table 4; Figure 4).

Table 4 indicates that ESTs encoding all five subtypes of histones, minichromosome-maintenance proteins and cell-proliferating nuclear antigens were among the transcripts whose expression was higher in the SAM than in the NM. The expression of these genes is known to be associated with DNA synthesis and cell proliferation, and they are thus abundant in the meristematic tissue [29,30] since this region consists of actively dividing cells.

In the same category, there is a transcript predicted to encode HIGH MOBILITY GROUP (HMG) protein. HMG are proteins that are known to play an architecture role in modifying DNA conformation to facilitate the assembly of multiprotein-DNA complexes. They may serve only to maintain physical orders but the involvement of these proteins in the network regulating SAM activity seems plausible. This is in view of recent studies that demonstrate the binding of HMG proteins to functionally important regions of plant gene promoter and stimulate transcriptions [31].

Other up-regulated sequences included transcripts predicted to encode cyclin D (AB008188) and cyclin B (EX570164), which are involved in the progression of the cell cycle. D-cyclins are one of the main rate-limiting factors for cell proliferation, and several of them may play key roles in the association between the cell cycle and meristem function, in particular primordia formation [reviewed in [32]].

### Epigenetic regulation of the SAM activity

Increases in transcript abundance were also found for genes predicted to encode histone-modification protein (EX569897), chromatin remodelling factors (EX570366, EX570306 and DQ026703) and DNA methyltransferases (EX568764 and AF034419; Table 4). These proteins are known to play a role in the epigenetic regulation of gene expression by participating in mechanisms that alter chromatin structure so as to activate or repress particular sets of genes [reviewed in [33]].

There is an emerging recognition of the significance of the chromatin remodelling process in regulating the activity of plant stem cells [reviewed in [34]]. For instance, mutation of the *Arabidopsis FASCIATA1 *(*FAS1*) and *FASCIATA2 *(*FAS2*) genes that encode subunits of the chromatin assembly factor leads to dysfunction of the SAM [35]. This was associated with the down-regulation of *WUS *gene expression in both mutants, suggesting that regulation of meristem function and organogenesis by chromatin remodelling factors is primarily achieved through regulation of the expression of the homeobox transcription factor [35]. However, direct links between chromatin remodelling factors and the regulation of the expression of key meristem genes remain to be established. Nonetheless, the up-regulation of the expression of these sequences in the SAM suggests that the mechanism of epigenetic regulation is important to maintaining the identity of stem cells in plants, as has been reported in animals [36].

### Transcription factors and hormonal regulation of SAM activity

We also identified a few putative transcription factors among the genes whose expression was higher in the SAM than in the NM. These included an EST (EX570025) annotated as a putative plant-specific transcription factor from a YABBY-family protein – members of this family are reportedly involved in the abaxial cell-fate specification in lateral organs of *Arabidopsis *[37]. Also on the list was a sequence predicted to encode AUXIN RESPONSE FACTOR 9 (ARF9; EX570238). Similar members of the auxin response factor group of proteins are known to regulate auxin-mediated transcript activation or repression. For example, the expression of several genes, such as those encoding members of LATERAL ORGAN BOUNDARIES domain proteins and AUXIN-REGULATED GENE INVOLVED IN ORGAN SIZE, are disrupted in the double mutant of ARF7 and ARF19, implicating their roles in auxin-mediated plant development [38].

Other gene products that might be related to auxin include a putative auxin-efflux carrier (PIN1), auxin-conjugating protein (DWARF IN LIGHT1) and a ribosomal protein L24B (Additional File 1, Table 4). Similar genes in *Arabidopsis *have been implicated in auxin-mediated developmental regulation [39-41]. For example, DWARF IN LIGHT1 is involved in auxin signal transduction, and inhibits shoot and hypocotyl-cell elongation [39]. The presence of several auxin-related transcripts on our list reflects the well-established roles of auxin in organ initiation and positioning at the meristem [42,43].

Intriguingly, miRNAs that are endogenous 21-nucleotide riboregulators have been shown to target several mRNAs implicated in auxin responses, including DWARF IN LIGHT1 [44]. There is increasing amount of evidence indicating that miRNA-mediated repression plays an important role in the spatial expression of plant cell-fate regulatory genes [e.g. [45]]. A protein called ARGONAUTE is known to function as a catalytic component of the RNA-induced silencing complex, which targets mRNA for degradation using miRNA as a guide [46]. Nevertheless, the precise role for the up-regulation of a transcript annotated as encoding a similar ARGONAUTE in our dataset (Table 4) awaits further study.

### Stress responses in the SAM

Many of the down-regulated transcripts were potentially associated with biotic and abiotic stress responses (Table 5), including transcripts predicted to encode dehydrin-related protein (AY065655), pathogenesis-related protein (AJ586324), disease-resistance-response protein (AF139018), antimicrobial defensin (AF525685) and chitinase (AB037832). This might be attributable to the stems, leaves and roots generally being exposed to greater biotic or abiotic stress than the well-shielded SAM, with the former therefore requiring the constitutive presence of these gene products at a higher level than in the SAM in order to maintain successful defence responses. However, we found at least one other sequence (EX571142) potentially related to stress responses whose expression was higher in the SAM (Table 4). Although the molecular basis for this is unknown, it is possible that the corresponding encoded product plays dual roles in both stress responses and development. This is supported by a recent study finding a network of rice genes associated with stress responses and seed development [47].

**Table 5:** Representative transcripts that are detected to be significantly down-regulated in the pea shoot apical meristem (SAM) in comparison to the non-meristem (NM) tissues.

**Probe^a^**	**Fold Change**	**Annotation^b^**
**Stress responses**		
AY065655	0.1	Ultraviolet B-repressible dehydrin-related protein
AY065659	0.1	Ultraviolet B- inducible protein
AJ586324	0.1	Putative basic PR1 protein
EX568748	0.3	Dehydrin
AF139018	0.3	Disease resistance protein
AF525685	0.4	Antimicrobial defensin
AJ278699	0.5	Protease
AB087832	0.4	Class 1 chitinase
AF175278	0.7	Wound-inducible P450 hydrolase
AF137351	0.2	Pathogenesis-related protein 4
**Reactive oxygen species homeostasis**		
EX568770	0.3	Catalase
AB026253	0.5	Copper amine oxidase
AJ50832	0.4	Germine-like protein
AB189165	0.4	Copper zinc superoxide dismutase
AB087837	0.7	Glutathione-S-transferase
AJ319808	0.3	Thioredoxin H
AB087838	0.5	Peroxidase
**Photosynthesis**		
EX569880	0.1	Light harvesting chlorophyll a/b binding protein
AY845255	0.2	Light harvesting chlorophyll a/b binding protein 3
EX569551	0.2	Light harvesting chlorophyll a/b binding protein type 1 (CAB)
AY292531	0.1	Oxygen-evolving enhancer protein
EX569675	0.3	Type II chlorophyll a/b binding protein
EX569989	0.4	PSI-K subunit of Photosystem I
AY007467	0.4	Photosystem II CP47 protein
EX570832	0.1	Ribulose 1,5-biphosphate carboxylase
AY065656	0.2	RUBISCO activase
**Metabolism**		
AY112702	0.6	Vacuolar acid invertase
AJ012080	0.3	Sucrose synthase
Y08728	0.6	ADP-glucose phosphorylase
EX569667	0.4	Ribulose-5-phosphate-3-epimerase
EX570956	0.7	Phosphofructokinase
**Cell wall modification**		
AF056493	0.7	Pectin methylesterase
AB042531	0.2	Xyloglucan endotransglycosylase
EX569643	0.6	Cellulose synthase
AJ621355	0.4	KORRIGAN
AB015428	0.6	Endoxyloglucan transferase 1
**Membrane- or transport-related**		
AJ243307	0.1	Putative plasma membrane intrinsic protein
AJ243309	0.3	Putative tonoplast intrinsic protein
AB027616	0.5	Apyrase
AF109922	0.7	Sucrose transport protein
EX570713	0.6	Aquaporin-like transmembrane protein
EX570839	0.7	Outward-rectifying potassium channel
EX569706	0.7	Sulfate transporter
**Unclassified/Unknown/No BLASTX match**		
AY371200	0.2	Ripening-related protein
AF369889	0.5	Embryo-abundant protein
EX568773	0.5	Hypothetical protein
EX569332	0.6	Hypothetical protein
EX569635	0.5	No BLASTX match
EX570920	0.7	No BLASTX match
AF515795	0.1	Dormancy-associated protein 3

a, Probe with GenBank accession number that begins with EX is derived from this study.

b. Annotation is based on the expect value of BLASTX hit that is set at 1e-5

### ROS homeostasis in the SAM

Surprisingly, the down-regulated transcripts included various sequences encoding proteins that scavenge or generate ROS, such as thioredoxin (AJ319808), catalase (EX568770), Cu-Zn superoxide dismutase (AB189165), copper amine oxidase (AB026253) and peroxidase (AB087838). This implies that the level of ROS is lower in the SAM than in the NM, and hence the expression of genes encoding ROS scavengers is lower in the former. The absence of photosynthesis (a source of ROS) in the SAM might explain the lower ROS level therein. However, there is increasing evidence that plants use ROS as signalling molecules for regulating development and various physiological responses, and for mediating abscisic-acid-induced stomatal closure, as well as in auxin signalling and gravitropism in roots [48]. Whether this indicates that the regulatory role of ROS is less prominent in the SAM than in the NM awaits further investigation.

### Photosynthesis- and cell-wall-related transcripts

Several of the genes more abundant in the NM than in the SAM were predicted to be related to photosynthesis, including subunits of photosystem I and II and chlorophyll-a/b-binding protein. It is well known that meristematic cells do not contain differentiated plastids, which may explain the lower expression level of genes associated with photosynthesis in the SAM.

Some of the sequences predicted to encode products that play roles in cellulose synthesis, such as cellulose synthase (EX569643), pectin methylesterase (AF056493), xyloglucan endotransglycosylase (AB042531), were found to be down-regulated in the SAM. This probably reflects the cell-wall structure in the stem and leaf being more complex that the thin primary cell wall in meristematic cells [4].

## Conclusion

The development of our EST collection from the pea SAM represents an important advance towards understanding SAM function and maintenance, especially due to the under-representation of SAM-related transcripts in the public database as demonstrated in this study. Subsequent transcriptional profiling experiments using the microarray constructed from ESTs yielded the transcriptional signatures of the pea SAM, and we have reported a repertoire of transcripts with putative or unknown functions that are differentially regulated in the SAM. *In silico *analysis of the predicted gene products has implicated several processes in the complex molecular network that regulates this developmental process. Future studies of these genes should attempt to reveal how they interact in the complex molecular network that maintains and regulates the dynamics of the SAM.

## Methods

### Plant materials and cDNA libraries synthesis

**Figure 5:**
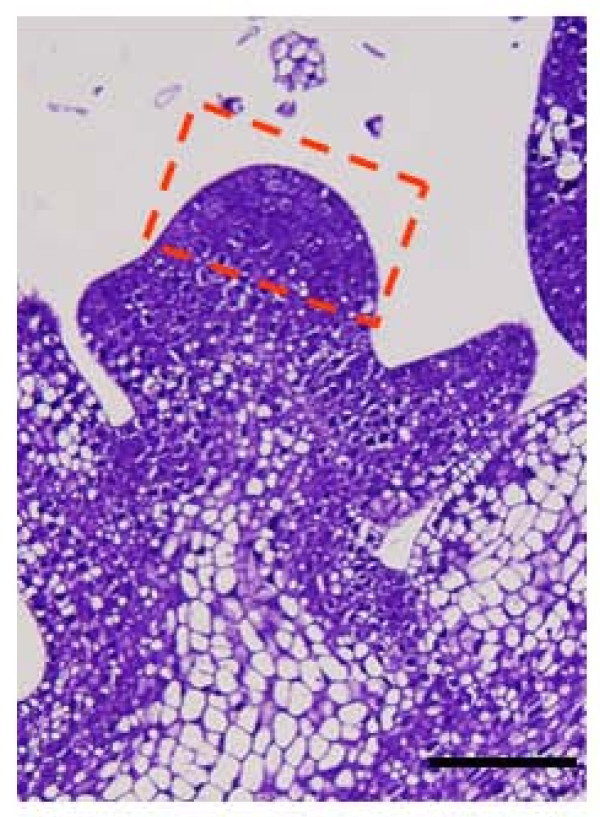
**Sampling strategy for the pea SAM.** Micrograph of a representative pea SAM, with the box showing the location of the tissue sample. Scale bar = 100 μm.

Garden pea (*Pisum sativum*) cultivar Torsdag was grown in a greenhouse located at the University of Melbourne, Australia. SAMs were micro-dissected from 10-day-old peas under the dissecting microscope at 40× magnification. Any leaf primordia were excluded in order to create a meristem-enriched tissue collection and the location of tissue sample is indicated in Figure 5. Dissected samples were quickly frozen in liquid nitrogen and stored at -80°C until used for RNA extraction and cDNA library synthesis [49]. For the subtracted library, the driver sequences were derived from an equal mix of RNA extracted from non-meristematic (NM) tissue consisted of primary stem (without axillary meristems), mature leaf lamina, primary roots (without root apical meristem and root hairs), whereas the tester sequences consisted of RNA harvested from dissected SAM. All cDNAs were cloned into pBlueScriptIISK+ plasmid vector.

### EST sequencing

A total of about 500, 1000 and 1500 randomly picked clones from each of the standard (C), subtracted (S) and normalized (N) libraries were sequenced at Australian Genome Research Facility (AGRF), Australia and subsequently at Macrogen Korea using T7 primer. These sequences have been deposited in GenBank under the accession numbers EX568682 to EX571416.

### Sequence analysis and annotation

Sequence data were trimmed off vector, adaptor and low quality sequences using SEQTools [50]. Trimmed sequences that were shorter than 100 basepairs were excluded from further analysis. Blast score-based clustering method with a score cut-off of 0.6 from SEQTools was then used to assemble the sequences. All clusters and singletons resulting from this automated clustering were considered to be the best estimation of a minimal gene set for our EST library and we have called this set as "unigenes".

All sequences were then imported into Blast2GO, a web-based Gene Ontology (GO) annotation and analysis tool [21] for subsequent analysis. This involved automated retrieval of GO terms associated to the hits obtained after a BLASTX search of the corresponding unigene sequence against NCBI (nr) protein database. The e-value cut off was set at 1e-5.

### RNA Extraction for microarray experiments

Total RNA was extracted from dissected SAM (approximately 80 SAMs per extraction) or other plant parts (primary stem, primary roots and mature leaves) using Qiagen RNeasy Mini Kit. Four independent tissue collections and RNA extractions (designated A, B, C and D) were performed for each of the microarray hybridization experiment.

### Design of pea SAM Combimatrix CustomArray™ 4X2K

The Combimatrix arrays are semiconductor-based oligonucleotide microarrays and are generated based on CombiMatrix technology (hyperlink) of *in situ *synthesis [51]. The CustomArray™ 4x2k is a microarray that is divided into 4 sectors, each of which can contain up to 2,240 different oligonucleotide probes (spots) and can be hybridized individually with different targets using a provided sectored hybridization cap. A total of 1686 pea sequences (290 sequences from C library, 300 sequences from S library and 1086 ESTs from the N library) together with 500 pea sequences randomly selected from GenBank pea protein database were submitted for probe design using the open source CombiMatrix probe design system. The length of probes ranged from 35–40 bases in length. A variety of control elements were also arrayed on the slide and these include blank spot, housekeeping genes (actin) as well as non-plant transgenes.

### Target preparation and hybridization to microarray

Target preparation and hybridization were performed in Australian Genome Research Facility Ltd (AGRF) according to the standard CombiMatrix protocol described in detail at . One microgram of total RNA from SAM or NM was labelled using the Kreatech's ULS™ RNA ampULSe kit to generate Cy5 or Cy3 labelled targets. The Cy5- or Cy3-labelled cDNA was then hybridized to different sector of the chip according to a balanced block design dual label experiment scheme [23]:

Sector 1: Cy3-SAM A vs Cy5-NM A

Sector 2: Cy5-SAM B vs Cy3-NM B

Sector 3: Cy3-SAM C vs Cy5-NM C

Sector 4: Cy5-SAM D vs Cy3-NM D

### Image acquisition and data analysis

The Cy5- and Cy3-hybridized chip was then scanned using Genepix 4000B microarray scanner (Axon Instruments, CA, USA) according to manufacturer's instructions. The generated tiff image files were then imported into Combimatrix Microarray Imager to produce intensity data. The LimmaGUI software, which is an implementation of the Empirical Bayes linear modelling approach, was used for subsequent statistical analysis of the resulting data [24]. A robust spline method was chosen for within array normalization and a least-square linear model fit was computed with the p-value adjusted using the Benjamini-Hochberg procedure. The entire differentially-expressed transcripts (p value < 0.05) in the SAM are listed in Additional File 1 and Additional File 2. Microarray data have been deposited in the Gene Expression Omnibus database [20] under accession number GSE9278.

### RT-PCR analysis

The one-tube, two enzyme Access RT-PCR system (Promega, Annandale, New South Wales, Australia) was used according to manufacturer's instructions in all RT-PCR analysis. Ten ng of RNA isolated from the SAM, mature stem, mature leaf and primary root of 10-day-old pea seedlings were used as a template in a 10 μl reaction volume. The pea actin gene was used as an internal control. The number of cycles used for the transcripts investigated was routinely between 25–28 and 80 % of the PCR reaction was separated on 1% agarose gel containing 0.1 μg/μl ethidium bromide and visualized under UV light.

## Abbreviations

SAM: shoot apical meristem; NM: non-meristem; EST: expressed sequence tag.

## Authors' contributions

CEW carried out the EST analysis, participated in the microarray experiment and RT-PCR analysis, and drafted the manuscript. PLB and MBS were responsible for design of the project, standardisation & organization of meristem micro-dissection & making EST libraries as well as overall coordination of the experiments and manuscript editing. HO contributed to EST library characterization and EST sequence analysis. All authors read and approved the final manuscript.

## Supplementary Material

Additional file 1 Transcripts identified to be up-regulated in the pea shoot apical meristem (SAM) in comparison to the non-meristem (NM) tissues. Click here for file

Additional file 2 Transcripts identified to be down-regulated in the pea shoot apical meristem (SAM) in comparison to the non-meristem (NM) tissues. Click here for file
